# Automatic Segmentation and Cardiac Mechanics Analysis of Evolving Zebrafish Using Deep Learning

**DOI:** 10.3389/fcvm.2021.675291

**Published:** 2021-06-09

**Authors:** Bohan Zhang, Kristofor E. Pas, Toluwani Ijaseun, Hung Cao, Peng Fei, Juhyun Lee

**Affiliations:** ^1^Joint Department of Bioengineering, University of Texas (UT) Arlington/(UT) Southwestern, Arlington, TX, United States; ^2^School of Optical and Electronic Information-Wuhan National Laboratory for Optoelectronics, Huazhong University of Science and Technology, Wuhan, China; ^3^Department of Electrical Engineering and Computer Science, University of California, Irvine, Irvine, CA, United States; ^4^Department of Medical Education, Texas Christian University (TCU) and University of North Texas Health Science Center (UNTHSC) School of Medicine, Fort Worth, TX, United States

**Keywords:** U-net, LSFM, segmentation, zebrafish, cardiac mechanics

## Abstract

**Background:** In the study of early cardiac development, it is essential to acquire accurate volume changes of the heart chambers. Although advanced imaging techniques, such as light-sheet fluorescent microscopy (LSFM), provide an accurate procedure for analyzing the heart structure, rapid, and robust segmentation is required to reduce laborious time and accurately quantify developmental cardiac mechanics.

**Methods:** The traditional biomedical analysis involving segmentation of the intracardiac volume occurs manually, presenting bottlenecks due to enormous data volume at high axial resolution. Our advanced deep-learning techniques provide a robust method to segment the volume within a few minutes. Our U-net-based segmentation adopted manually segmented intracardiac volume changes as training data and automatically produced the other LSFM zebrafish cardiac motion images.

**Results:** Three cardiac cycles from 2 to 5 days postfertilization (dpf) were successfully segmented by our U-net-based network providing volume changes over time. In addition to understanding each of the two chambers' cardiac function, the ventricle and atrium were separated by 3D erode morphology methods. Therefore, cardiac mechanical properties were measured rapidly and demonstrated incremental volume changes of both chambers separately. Interestingly, stroke volume (SV) remains similar in the atrium while that of the ventricle increases SV gradually.

**Conclusion:** Our U-net-based segmentation provides a delicate method to segment the intricate inner volume of the zebrafish heart during development, thus providing an accurate, robust, and efficient algorithm to accelerate cardiac research by bypassing the labor-intensive task as well as improving the consistency in the results.

## Introduction

Biomechanical analysis is vital during cardiac development, as assessment of biomechanics is closely associated with regulation of valve formation, ventricular septum, and trabecular morphology related to cardiogenic transcriptional and growth/differentiation factors (1, 2). Lack of intracardiac biomechanical force could induce genetic programming's malfunction resulting in congenital heart defects in humans and mice (3). For example, understanding potential malfunctions within the heart's subcellular structure could point toward indications of different maladies such as ischemic heart disease (IHD). Severino et al. (4) reported that IHD is associated with coronary microvascular dysfunction, which is affected by the role of ATP-sensitive potassium channel, and this ATP-sensitive potassium channel modulates the degree of contractile tone in vascular muscle (5). Thus, investigating biomechanics to link disease models including contractility is an important assessment in cardiac research field.

Volume change-based cardiac mechanics measurements (e.g., ejection fraction) from the complex trabeculated and beating heart are most commonly used and play an essential role in evaluating the cardiac health condition. Such measurement relies on the accurate reconstruction of the heart's volume, which depends on the accurate segmentation of the biomedical images. Although the segmentation of biomedical images for volume reconstruction has been extensively studied in radiation imaging techniques such as MRI or CT (6–8), these imaging methods are challenging to be adopted for optical fluorescent images. Although various optical microscopes have been extensively used to study in biomedical research due to inexpensive approach, high resolution, and amenable fluorescent tagging (9–11), it suffers from light scattering and different intensity of the fluorescent signal. In addition, imaging dynamic samples is another challenge for microscopes. Unlike conventional microscopes, however, light-sheet fluorescent microscopy (LSFM) circumvents these challenges to capture *in vivo* dynamic samples, such as zebrafish heart, with a high axial resolution, deep axial scanning, fast image acquisition, and low photobleaching (12, 13).

Despite having a two-chambered heart and a lack of a pulmonary system, the zebrafish represents an emerging vertebrate model for studying developmental biology (14, 15). Its transparency and short organ developmental timeline enable rapid and high throughput analysis of developmental stages with optical fluorescent technology (16). Such advantages of zebrafish and LSFM systems make a powerful tool for studying *in vivo* cardiac development.

To understand the cardiac function and mechanics and further analysis, intracardiac segmentation is a necessary step (2, 17). Previously, segmentation of the LSFM images for measuring cardiac mechanics was accomplished manually by recognizing different intensities from large amounts of samples or tissue scatterings engenders many variables (18). The time-consuming task of manually segmenting the LSFM images is infeasible when processing high axial resolution data, as the number of images required is enormous (19, 20). On the other hand, lower axial resolution degrades the volume measurement's accuracy, while inconsistent manual segmentation poses a threat to the cardiac mechanic analysis' overall quality. Recently, Akerberg et al. (21) nicely demonstrated SegNet-based deep-learning segmentation of zebrafish hearts at the early ventricular developmental stage before trabeculation. However, accurate segmentation of complex ventricular morphology after initiating trabeculation is critical to cardiac mechanics analysis. Therefore, we utilize the advancements in a specific convolution neural network (CNN) architecture, namely, U-net (22), which performs binary classification of the LSFM images' pixels. Unlike natural images, in which rich color and texture information are provided, LSFM images offer limited information. To reliably segment an LSFM image, the pixel location has to be considered. Our proposed U-net utilizes such information via a multiscale processing pipeline, which uses downsampling to extract the features while using upsampling to convert features to specific pixel positions. The main contribution of this paper is to propose and demonstrate a practical and robust U-net architecture for optical imaging, which is tailored to segment LSFM images of the developing zebrafish heart. The synchronization of the LSFM image sequence is applied before segmentation as a preprocessing step.

For our application, the U-net was trained to utilize LSFM images of a zebrafish during ventricular development from 2 to 5 days postfertilization (dpf). In this paper, we explore the potential use of the U-Net architecture to expedite the segmentation of the intracardiac zebrafish heart, including the atrium and ventricle and further biomechanical analysis of the extracted results from the network.

## Methods

### Zebrafish Preparation for Imaging

The zebrafish used for this study was raised and maintained in our zebrafish core facility under the required UT Arlington Institutional Animal Care and Use Committee (IACUC) protocol. Transgenic *tg*(*cmlc2:gfp*) zebrafish lines were used in this study to observe myocardium and chamber development. To ensure a clear image, a medium composed of 0.0025% phenylthiourea (PTU) was used to suppress pigmentation at 20 h postfertilization (hpf) (23). Before imaging, zebrafish embryos were anesthetized in 0.05% Tricaine and immersed in a solution of 0.5% low-melt-agarose at 37°C. The embryos were then transferred to a fluorinated ethylene propylene (FEP) tube (refractive index, 1.33) to minimize refraction along the path of light before image acquisition. This FEP tube was then immersed in water (refractive index, 1.33) and connected to an in-house LSFM to scan 500 images per slice from the anterior to posterior of zebrafish heart with 2 μm thickness (24).

### 4D Reconstruction of Beating Zebrafish Heart

4D reconstruction of *in vivo* beating zebrafish heart was performed using previously described methods (18, 25). Performed 4D reconstruction procedure is based on assumption that zebrafish heartbeat is regular throughout the image acquisition. However, experiments demonstrate that this assumption is not reliable. To minimize the natural irregularity of zebrafish heartbeat, we added an extra parameter (δ) to detect phase lock at period determination to observe irregularity during the experiments (18, 25). This latent variable window, δ, can be set roughly −0.3 to +0.3 ms after finding the estimated cardiac period manually (26).

### Segmentation of Intracardiac Domain

Well-reconstructed 4D images were selected for manual segmentation of the intracardiac domain. 3D images of each time point from the beating heart were loaded into the Amira software (Thermofisher Scientific, Waltham, MA) or 3DSlicer. First, the inner cavity of each 2D slice was carefully selected manually and reconstruct 3D volume segmentation for each time point of the beating heart. We repeated this process at all developmental points to make segmentation masks. Manual segmentation masks were used for training our U-net architecture.

### Application of U-Net Architecture

**Figure 1:**
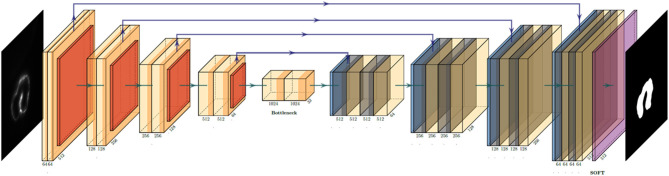
U-net convolution neural network (CNN) architecture utilized to generate the binary mask of the intracardiac domain of zebrafish. Each box represents a multichannel feature map that allows for efficient and accurate extraction of anatomical features. In our specific application, the input was a 512 × 512 pixel map.

The U-net architecture revolves around two distinct paths: the contracting path and the expansive path (22). The network takes the input of size: A × A × n {A = 2k|k∈N} array, where “n” is the color channel or the depth of image. Since our LSFM provides a grayscale image of singular depth, “n” was 1. The input is pushed initially through the contracting path, which processes the input through a series of 3 × 3 convolutions, the application of a rectified linear unit (ReLU) following each convolution, then a 2 × 2 max-pooling operation. This yields an output of A/2 × A/2 × 2n, expanding the feature channel by double. This process is repeated four times before the final output is started to processed by the expansive path, which undergoes a similar process, with the exception of using a 2 × 2 convolution operation instead of max pooling (Figure 1). The loss function used for this particular project was a binary cross-entropy loss function, and the Adam optimizer was used. The computer used was an Intel Xeon E5 (CPU) and NVIDIA Quadro P5000 (GPU). Most of the calculations were done by GPU; it consumes 3.39 GB of memory, and performance scales with more memory allotted. With the current setup, it costs around 180 ms to train each slice; the minimal request for training dataset (400 slices) and epoch number (50 epochs) would be 48 minutes. It takes about 60 ms to predict one slice. In a few minutes, the network can process the whole 4D data.

### Network Training With Manually Segmented Images

Training data for CNN learning is divided into two separate bins: training volumes and labels. Four individual heart samples models were used to study the developmental stages from 2 to 5 dpf. The raw data came from 4D images of the beating heart. From these 4D images, each 3D images were isolated from every 20 equally spaced sample of each heart. For these, we selected the entire slices of the 20 samples of 3D volume of the heart from 4D dataset, representing three cardiac cycles, with each volume containing m numbers of 2D sliced images (m∈N), which was dependent on the slice selected. This particular parameter was related to the spatial depth of the volume being observed. Following data acquisition, hand segmentation was performed using 3DSlicer GUI application on the 2D axial plane spanning the entirety of the zebrafish heart captured. Both of these training bins were converted into 8-bit format and imported into U-NET as a 512 × 512 × 1 8-bit array, with bijective correspondence between volumes and labels. The U-NET program featured trained blind to the spatial depth of the volume itself, only having access to segmentation of the 2D axial slice input. From here, the training data took up approximately 80% of the data used for experimentation, while 20% was used for future validation.

### Dice Similarity Coefficient Correlation

The primary method to which our automatic segmentation was assessed was using the Dice similarity coefficient correlation, which compares the amount of space of which the volumes of the automatic and hand segmentation overlap in comparison with the summation of the total number of pixels. The calculation of this is as follows for area A, representing autosegmentation, and area B, representing our manually labeled segmentation:

(1) $$\begin{array}{l} \text{Similarity}\;=\;\frac{2\times |\left(A\cap B\right)|}{\left|A|+|B\right|} \end{array}$$

The outcome yields some value *x* for *x* ∈ [0, 1], with the value 1 (approximating a near-perfect segmentation match with respect to the initial hand segmentation used) and 0 (showing essentially no correlation between the two).

### Cardiac Mechanics Analysis

After segmented volume, we multiplied voxel resolution to obtain the actual volume of zebrafish heart. We capture the size of most dilated points and most contracted points as end-diastolic volume (EDV) and end-systolic volume (ESV), respectively. Stroke volume (SV) was calculated by subtracting ESV from EDV. Ejection fraction (EF) is the ratio of blood ejection, which can be simply calculated from EF = (SV/EDV) × 100.

## Results

### Manual Segmentation From LSFM for U-Net Training

**Figure 2:**
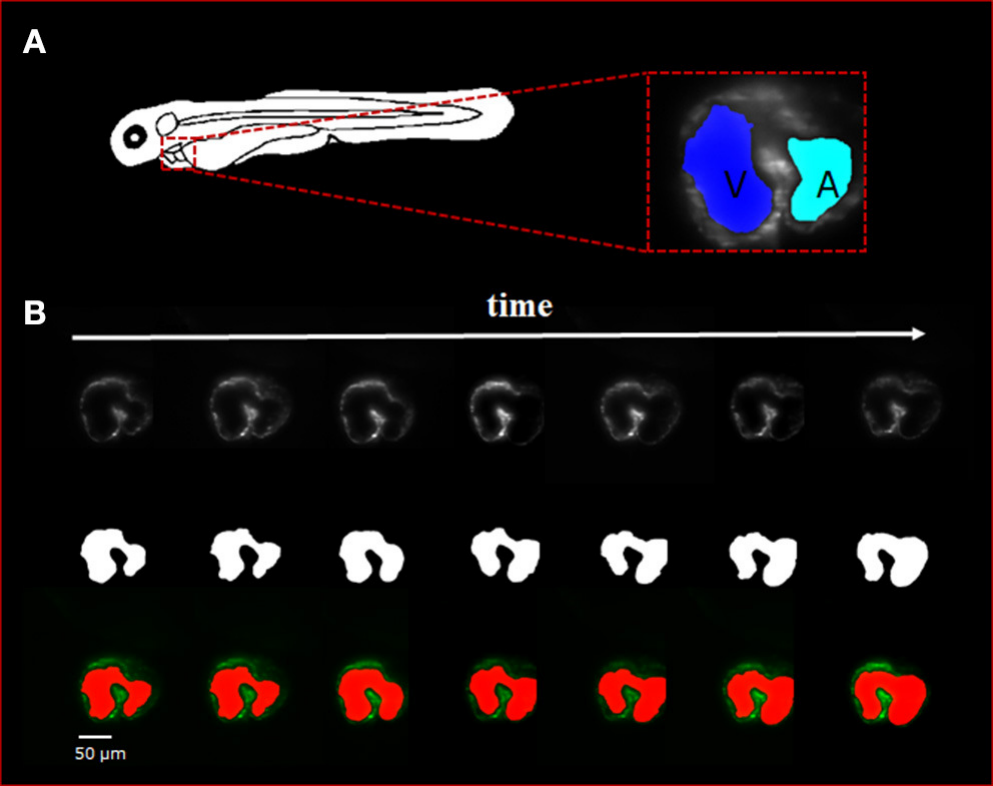
Sequence of selected light-sheet fluorescent microscopy (LSFM) images with the manual hand segmentation mask from 4 dpf zebrafish heart. **(A)** Diagram showing the anatomical feature of the zebrafish heart as well as a single axial slice with corresponding binary mask generated by the U-net. It is observed that there is a clear distinction between the atria and ventricle of the specimen. **(B)** A sequence of selected axial slices with corresponding binary masks generated by our U-net program; the reference scale bar is 50 μm.

Manual segmentation was performed along the 2D axial plane of the zebrafish heart for each studied sample. The methodology for manual segmentation, which served as our ground truth and labels, was performed using a contour-based segmentation method provided by 3DSlicer and was done in conjunction with *ad hoc* edits done on the resulting figures. Manually segmented images were used as training datasets for U-net (Figure 1). The concept of U-net architecture was to supplement a usual contracting network by successive layers, where pooling operators were replaced by upsampling operators (22). Here, original 512 × 512 pixels LSFM images were deconstructed to detect features of intracardiac boundaries. Then, upsampling of each layer increased the resolution of the output. For localization, higher resolution features from the downsampling path were combined with the upsampled output. As the manual segmentation images were input for training, a successive convolution layer learned to assemble a more precise output based on this information. In addition, a Gaussian 2D filter of kernel size of 2.0 μm was applied to smooth manual segmentation. Contour interpolation was further applied to the 3D structure output to minimize the step size of later 3D reconstruction. The fidelity of the manual segmentation was done observationally through inspecting the spatial comparison between the label generated and the intracardiac area of the 2D slice (Figure 2).

### Validation of U-Net Based Autosegmented Image

**Figure 3:**
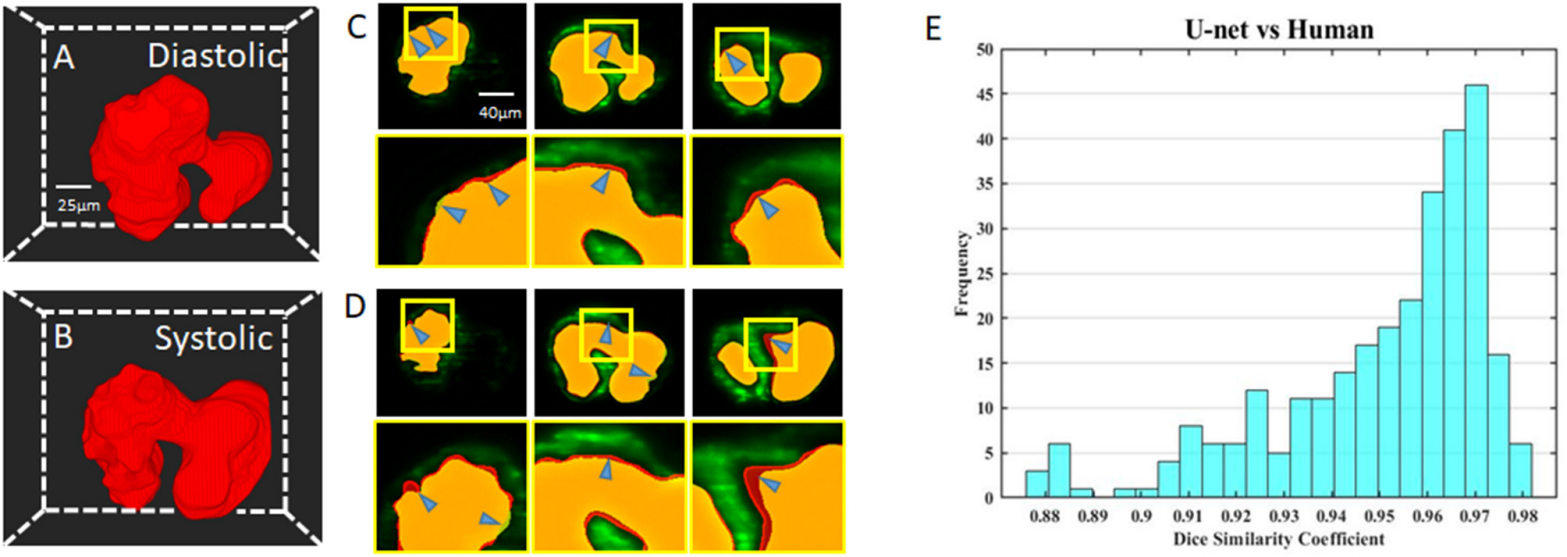
Comparison of U-net based autosegmentation to manual hand segmentation. **(A,B)** Geometric representation of both the diastolic and systolic stages of the heart cycle. **(C,D)** Comparison in the 2D axial plane between autosegmentation of the U-net and ground truth in both the diastolic and systolic stages, respectively. The blue arrows indicate areas of disjoint space within the segmentations, shown in red (manual segmentation), green (autosegmentation), and yellow (intersection). **(E)** Corresponding Dice similarity coefficient comparing the results between our ground truth and automatic segmentation demonstrated the ability of autosegmentation.

We visualize the segmentation ability of our networks by reconstructing the diastolic and systolic stages of zebrafish (Figures 3A,B; Supplementary Videos 1, 2). Although there is a small segmentation discrepancy between ground truth and autosegmentation around fluorescent boundaries (Figures 3C,D), U-net-based segmentation accuracy for each frame has a mean Dice coefficient score of 0.95 with a standard deviation of 0.02 (Figure 3E). Due to trabeculation in the ventricle at 4 dpf, manual segmentation was more sophisticatedly segmented in rough fluorescent boundaries. Although our autosegmentation was performed to detect rough boundaries in the trabeculated area, the roughness was relatively smooth. However, the atrium's smooth surface and innermost area of the ventricle were captured closer to the fluorescent signal. This discrepancy could be from the dataset that we used to train the network, the result of inconsistences from manual segmentation judged by the user. These statistics demonstrate our U-net-based segmentation's remarkable ability to segment intracardiac chamber with a high dice coefficient score and reduces inherent manual segmentation.

### Automatic Morphology to Separate Inner Chamber Space

To validate the U-net-based autosegmentation and allow for further application, it was deemed necessary to derive some function for separating the inner-chamber structure to allow for independent analysis of the two-volume components and their disjoint biomechanical characteristics. This was done using an in-house Matlab code utilizing an iterative process of applying an erosion operator relying on a 3D structuring element until fracturing the single master volume. Following this, a dilation operator was applied to the individual subvolumes to recover lost space, and the resulting dilated volumes were intersected in 3D space with the initial master volume. The output of this program was the resulting intersected space.

**Figure 4:**
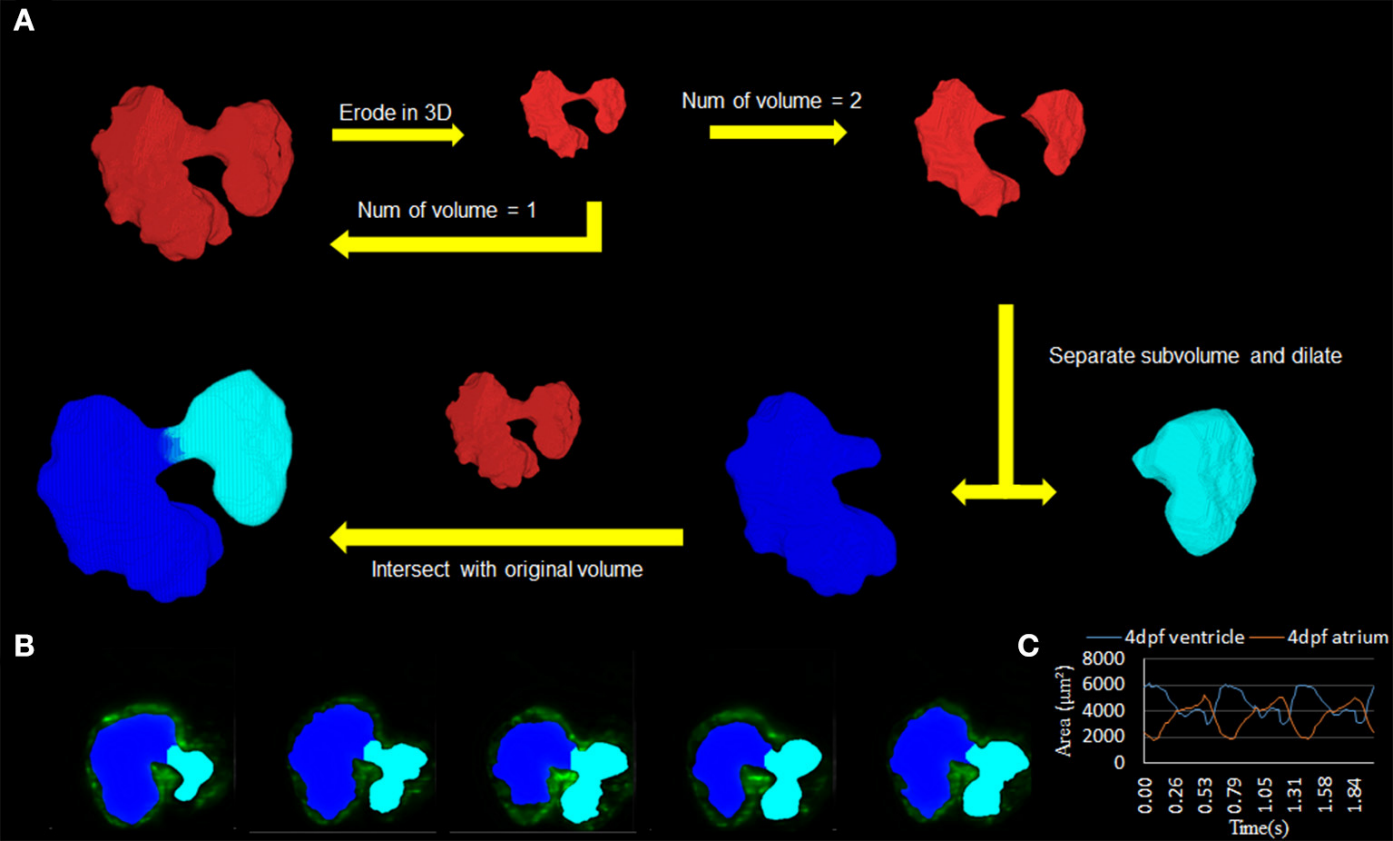
Subdivision of atrium and ventricle. **(A)** Methodology of using the automatic segmentation to define the morphology of inner volume of the zebrafish heart. This method relies on an iterative process that uses an erosion operator on the 3D structure of the inner volume until division occurs. Following the division of the volume into two separate components, extraction of each individual piece follows with a dilation operation and intersection with the original volume. **(B)** 2D axial slices showing the differentiation of the atrium (teal) and ventricle (blue) of various slices. **(C)** Area change over time from 2D slice **(B)** was successfully demonstrated.

This program's use proved to be critical in studying the resulting biomechanical elements of the atria and ventricle, as it allowed for consistency in determining the chambers (Figure 4A; Supplementary Videos 3, 4). The methodology was used successfully to distinguish the atrium and ventricle within the program on a three-dimensional level (Figure 4B). The resulting axial scans of the separated atria and ventricle show a clear distinction between the two different chamber areas per slice at 4 dpf (Figure 4C).

### Volumetric Analysis

**Figure 5:**
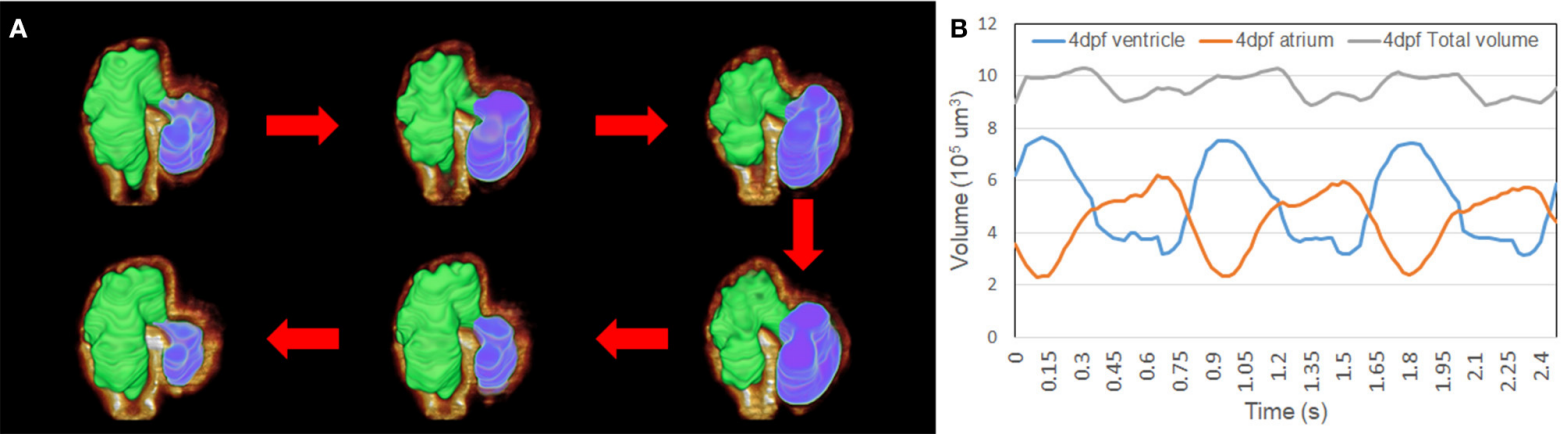
Representation of the contraction to dilation of zebrafish heart volume change over time. **(A)** Autosegmented successfully reconstructed 4D image captured the rough inner surface of zebrafish due to trabeculation after merging with fluorescent-labeled *tg*(*cmlc2:gfp*) zebrafish from light-sheet fluorescent microscopy (LSFM) images. **(B)** Volume change in the ventricle and atrium was measured from autosegmentation. Total volume of atrium and ventricle represented around 9.5 × 10^5^ μm^3^ when zebrafish was at 4 dpf.

We have applied 4D synchronization methods to reconstruct volume change over time (2, 18). Analysis of the volume change along three cardiac cycles was provided to study the biomechanical changes over time (Figure 5A). The volume of the atria and ventricle and the total intracardiac volume of both chambers were plotted independently (Figure 5B). Although the total cardiac volume of 4 dpf is oscillating during pumping, the mean value is roughly 9.5 × 105 μm^3^. At 4 dpf, trabeculae developed in the ventricle; thus, 4D reconstruction showed the motion of the corrugated surface of the ventricle. Due to the lack of trabeculae (27), the atrium has a relatively tranquil curvature motion during atrial contraction and relaxation. Our results reveal the potential power of complex morphology and functional analysis.

### Assessment of Cardiac Mechanics of Developing Zebrafish Heart

**Figure 6:**
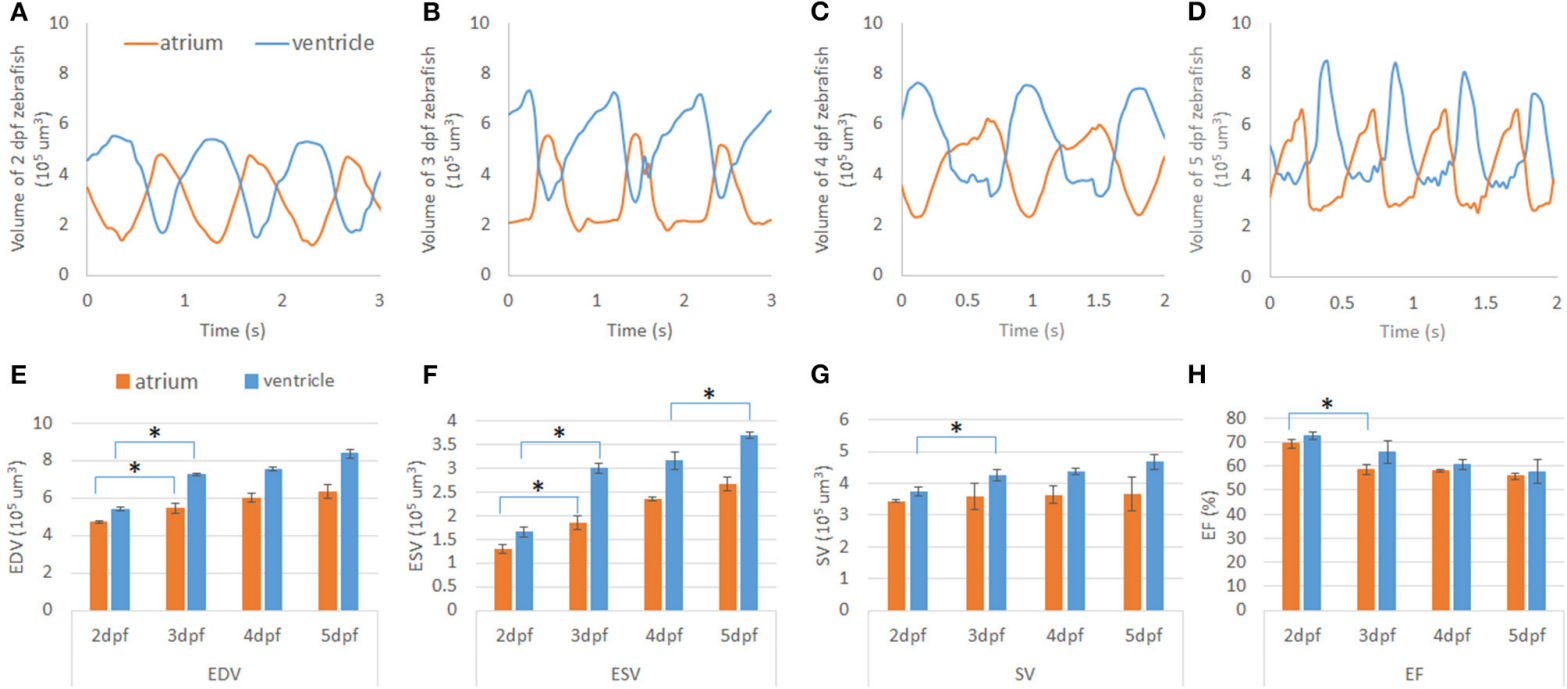
Cardiac mechanics analysis of developing zebrafish heart. **(A–D)** Volume change of developing zebrafish heart was measured by U-net-based autosegmentation, showing consistent increase in volume in both the atrium and ventricle. **(E,F)** After notable morphology change after cardiac looping, end-diastolic volume (EDV) and end-systolic volume (ESV) were increased significantly in both the atrium and ventricle. At 5 dpf, ESV was also significantly increased compared to 4 dpf. **(G)** While SV of the ventricle showed an increasing trend, that if the atrium remained at a similar level from 2 to 5 dpf. **(H)** Ejection fraction (EF) analysis demonstrated a decreasing trend from high EF at 2 dpf. *p ≤ 0.05.

In studying the volumetric change of the zebrafish heart during early-stage development between 2 and 5 dpf, observations allowed for the analysis of the atria and ventricle volumetric loads. Interestingly, we recognize that the ventricular contraction pattern shifts left from 2 to 3 dpf and shifts right from 4 to 5 dpf. Similarly, a pattern was observed within the atrium's volumetric change, albeit in an inverse manner (Figures 6A–D). Furthermore, based on volume change data, we have performed cardiac mechanics analysis during development. We first analyze the end-diastolic volume (EDV) and end-systolic volume (ESV) of the atrium and ventricle. Although the EDV and ESV trend of both the atrium and ventricle consistently increased, there was a significant increase between 2 and 3 dpf where morphology changed by cardiac looping (28) (Figures 6E,F). The active trabeculation process (18, 27), which increases the ventricle's contractility, affects the ESV of the ventricle at 5 from 4 dpf. Interestingly, the ventricle's stroke volume (SV) consistently increased, while the SV of the atrium remains consistent around 3.6 × 105 μm^3^ during early cardiogenesis (Figure 6G). We have further analyzed ejection fraction (EF) from EDV and ESV of the atrium and ventricle to understand how much blood each chamber pumps out with each contraction. Although, at 2 dpf, EF was high in both the atrium and ventricle, it continuously decreased throughout development (Figure 6H).

## Discussion

Our U-net-based segmentation methods successfully provided intracardiac 3D volume structure for studying the biomechanics of zebrafish hearts trained with manually segmented LSFM images. The previously created manual segmented volume was a tedious and taxing process for the researcher, leading to a possible room for error as time requirements increase. Furthermore, inconsistency would be induced between individuals, yielding different results of the same volume for two people. Although Akerberg et al. (21) used a SegNet-based autosegmentation nicely segmented zebrafish intracardiac volume and analyzed the cardiac function, their application was visualized in an early stage of zebrafish heart before trabeculation. We have adopted U-net for the segmentation to apply complex geometry of inner ventricular surface until 5 dpf zebrafish heart. This study relies on a fully convolutional neural network, which allows for quick segmentations with as little as five datasets. Within this experiment, our application revolved around pairing U-net with LSFM. This particular study's novelty was the new relation between the LSFM and U-net structure, which allows for high-level sectioning capabilities of the LSFM to generate the 3D structure of the complex zebrafish heart with ease, followed by automatic morphological analyses.

There are currently a few limitations to our approach from this paper, which we feel necessary to touch upon. A limitation to the algorithm is that it relies on manual segmentation as its ground truth. Furthermore, the quality of the manual segmentation also depends on the image quality of each raw LSFM image slice. If the raw images are perhaps not precise enough for various reasons, the algorithm may not be able to generate the most precise results it is capable of. As for manual segmentation, there is a high possibility of human error. There are many variables that change in the manual segmentation depending on the user. One aspect of this is smoothing of the 2D slices in the manual segmentation. The smoothing is done based on each user's discerning eye, which can lead to minor discrepancies in the manual segmentation. In addition, the data for the algorithm are trained separately by day instead of a single dataset, which limits efficiency due to the complex nature of the morphology and different developmental environment. As a consequence of the change in morphological structure observed over the time period of which this project was performed, it was not feasible to create some singular model that could automatically segment any 2D axial input for any developmental stage. As a consequence, we found it necessary to create four different models, one for each corresponding developmental stage for independent processing. This limitation required extra work in terms of computational requirements; however, we see it as necessary for the moment. Despite these limitations, the current algorithm designed has been able to create highly accurate autosegmentation from the use of manual segmentation of four different developmental datasets.

This study's primary objective was to determine if the conjunctional use of this modality-program pair provided feasible results. This objective was satisfied with a high degree of success, as seen from the Dice similarity correlation coefficient having a value perceived as exceptional, showing similarity between our perceived ground truth and the autosegmentation (Figure 3E). Although we have observed errors between manual and autosegmentation, we assume that the fluorescent intensity threshold point was vague when segmentation was performed manually (Figures 3C,D). Therefore, our autosegmentation network could help to process more detailed cardiac mechanical analysis compared to manual segmentation. Most interestingly, in our cardiac mechanics analysis, relatively rapid contraction and slower relaxation of the atrium at 2 and 3 dpf transitions to slower contraction and rapid relaxation at 4 and 5 dpf (Figures 6A–D). At 2 and 3 dpf, zebrafish atrium pumps blood in peristaltic motion due to lack of valves, while it relies on impedance pumping mechanism at 4 and 5 dpf (29). Therefore, we may observe that the atrial volume change curve shifts left at 2 and 3 dpf and shifts right at 4 and 5 dpf. In addition, after cardiac looping, cardiac function significantly changed as the ventricle developed faster in size. In the heart's tubular shape before cardiac looping, the atrium is bigger than the ventricle (21). At 5 dpf, when active trabeculation increases the ventricle's contractility, contraction of the ventricle increased significantly in corroboration with previous findings (Figure 6F) (18). Atrial SV stayed consistent during development in the interim of increasing SV of the ventricle (Figure 6G).

Despite rapid process and consistent results, the experiment conducted has some mild limitations when considering the overall capabilities being used. The most critical component for this study, including any deep-learning-based segmentation process, requires a high-quality input dataset, as the output test labels are only as good as those they are trained from. In our case, using the fluorescent label zebrafish, *tg*(*cmlc2:gfp*), yielded overall strong results from the ventricle but, on occasion, could have issues with the atria due to lower cardiomyocyte density and its location deeper in the chest, leading to more imaging issues. Circumventing this issue was not a trivial action, as exploring extensive preprocessing methods was required to be able to find the boundary of the inner volume of the heart (30). Other limitations that could be found in the experimentation process could be issues within the imaging process, in which user and systematic errors could hinder image fidelity.

Our methods will be compatible with other fluorescent optical imaging, such as z-scanned confocal microscopy images, providing quality 3D reconstruction. Our powerful U-net-based segmentation could be a new tool for studying a variety of future biomedical research applications. To begin, the parameters of the U-net could be tweaked to increase the accuracy and precision of the program for the zebrafish hearts; this would lead to a better quality autosegmentation, which would lead to progress in the field of mechanobiology by using computational fluid dynamics to understand the shear stress or pressure that could affect cardiac morphogenesis (2, 17).

## Conclusion

Our U-net-based segmentation provides a delicate method to segment the intricate inner volume of zebrafish heart during development, thus providing an accurate and convenient algorithm to accelerate cardiac research by bypassing the labor-intensive task as well as improving the consistency in the results.

## Data Availability Statement

The raw data supporting the conclusions of this article will be made available by the authors, without undue reservation. Our all source codes are available in our Supplementary Materials.

## Ethics Statement

The experiments were performed in compliance the approval from the UT Arlington Institutional Animal Care and Use Committee (IACUC) protocol (#A17.014).

## Author Contributions

BZ and KP: developing U-net for cardiac segmentation. KP and TI: image data acquisition and image processing for training. BZ, KP, and TI: training network and data analysis. HC, PF, and JL: conceptual design, research guide, and data interpretation. All authors edited and revised the article.

## Conflict of Interest

The authors declare that the research was conducted in the absence of any commercial or financial relationships that could be construed as a potential conflict of interest.
